# RiboGalaxy: A Galaxy-based Web Platform for Ribosome Profiling Data Processing – 2023 Update

**DOI:** 10.1016/j.jmb.2023.168043

**Published:** 2023-03-09

**Authors:** Alla D. Fedorova, Jack A. S. Tierney, Audrey M. Michel, Pavel V. Baranov

**Affiliations:** 1 School of Biochemistry and Cell Biology, University College Cork, Cork, Ireland; 2 SFI Centre for Research Training in Genomics Data Science, University College Cork, Cork, Ireland; 3 EIRNA Bio Ltd, Bioinnovation Hub, College Rd, Cork, T12 TP07, Ireland

**Keywords:** ribosome profiling, ribo-Seq, galaxy, mRNA translation, translatome

## Abstract

Ribosome profiling (Ribo-Seq) captures a “snapshot” of ribosomes’ locations at the entire transcriptome of a cell at sub-codon resolution providing insights into gene expression and enabling the discovery of novel translated regions. RiboGalaxy (https://ribogalaxy.genomicsdatascience.ie/), a Galaxy-based platform for processing Ribo-Seq data is a RiboSeq.Org (https://riboseq.org/) resource. RiboSeq.Org is an online gateway to a set of integrated tools for the processing and analysis of Ribo-Seq data. In this RiboGalaxy update we introduce changes to both the tools available on RiboGalaxy and to how the resource is managed on the backend. For example, in order to improve interoperability between RiboSeq.Org resources, we added tools that link RiboGalaxy outputs with Trips-Viz and GWIPS-viz browsers for downstream analysis and visualisation. RiboGalaxy’s backend now utilises Ansible configuration management which enhances its stability and jobs are executed within Singularity containers and are managed by Slurm, strengthening reproducibility and performance respectively.

## Introduction

Developed in 2009,^1^ Ribosome profiling (Ribo-Seq) is a method for obtaining quantitative information on the locations of the ribosomes translating RNA which has been widely used in various species.^2–6^ This information can help researchers determine which regions of RNA are being actively translated in a cell. Ribo-Seq has gained popularity due to its ability to provide insights on many aspects of the protein synthesis process, including translation initiation,^7–9^ speed of elongation^9–11^ and ribosome pausing^12–15^ to name just a few. Ribosome profiling has provided important insights into the regulation of translation and how it is modulated by various stress conditions.^16–21^ It is also a powerful tool for investigating drugs that interfere with translation, enabling unbiased identification of their targets and characterising the mechanisms of drug actions.^22–23^

During the ribosome profiling experimental procedure, fragments of mRNA that are protected from nuclease digestion by a translating ribosome are isolated and sequenced to produce samples of footprints typically ~30 nucleotides in length. For each ribosome footprint it is possible to retrieve the position of the first nucleotide of the A-site codon which corresponds to one of the three possible translated reading frames. This level of precision (which is also called sub-codon resolution) is what leads to the property of 3-nucleotide periodicity in Ribo-Seq data. Utilising the Galaxy Project’s open-source data processing framework, RiboGalaxy provides users with the means to process raw reads using a Galaxy interface, enabling robust and reproducible data analysis.

RiboGalaxy is part of a collection of resources for the processing, analysis and visualisation of publicly available ribosome profiling data called RiboSeq.Org. The other resources in this collection (GWIPS-viz^24–25^ and Trips-Viz^26–27^ allow users to explore and analyse preprocessed publicly available datasets or to upload their own data for visualisation and detailed inspection.

RiboGalaxy has been freely available to the public since 2016.^28^ It went through a complete overhaul recently taking advantage of recent Galaxy developments with respect to backend software architecture, Galaxy best practices and up-to-date source code. The updated version of RiboGalaxy is now available at https://ribogalaxy.genomicsdatascience.ie/.

## Results

RiboGalaxy is now built with more recent deployment best practices and has undergone multiple advances since the last publication (Table 1).

### Backend

In line with the Galaxy Project, RiboGalaxy’s backend now utilises Ansible, a configuration automation and package management tool which is more reproducible, easier to recover and manage than the original RiboGalaxy instance. Slurm is now used to schedule jobs^29^ and each job is now run in its own Singularity container.

### Tools

**Figure 1:**
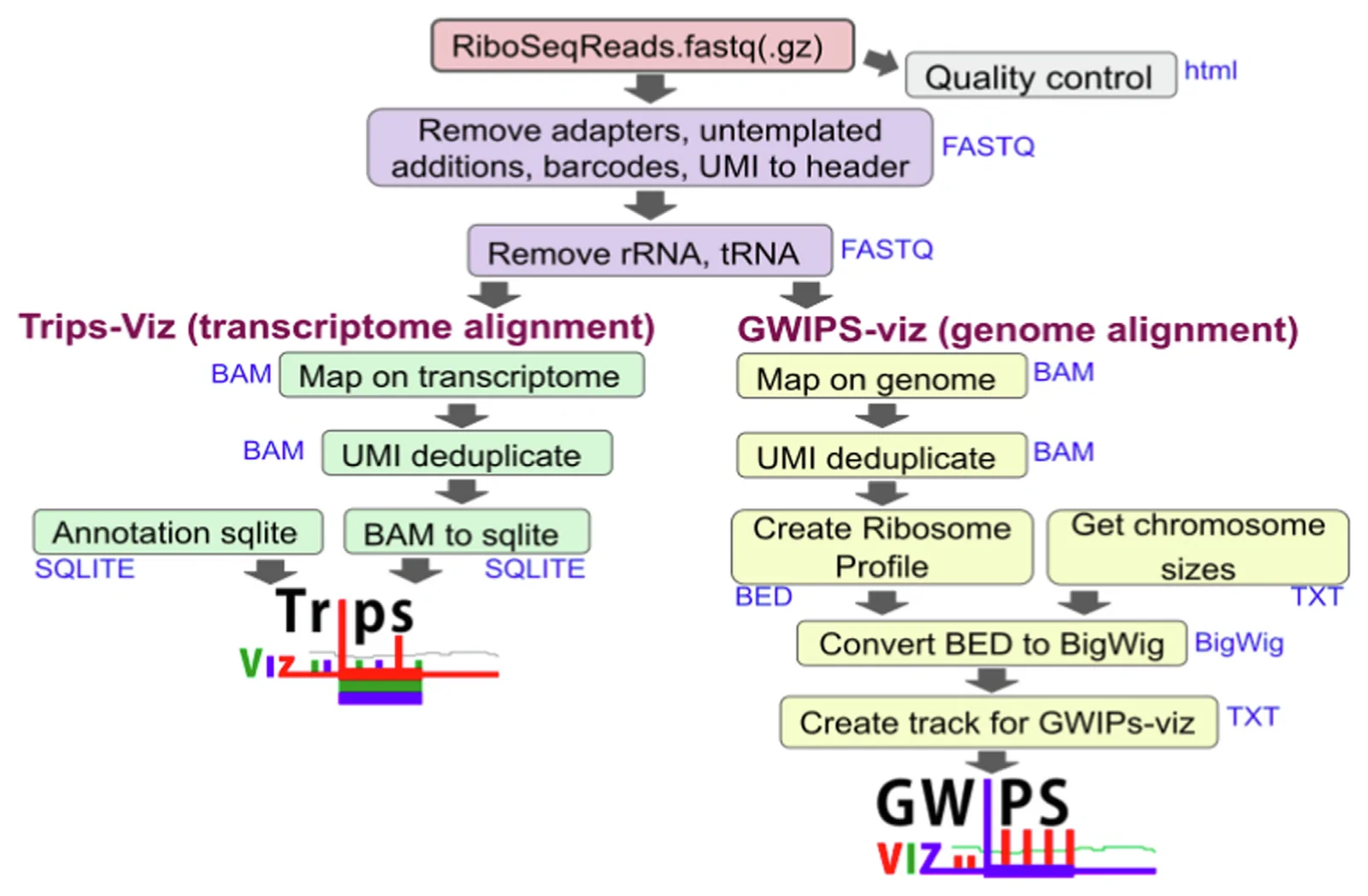
Schema of the typical simplified pipeline that can be run in RiboGalaxy. Input files containing raw reads (in this case - Ribo-Seq reads) can be either plain FASTQ or gzipped FASTQ. During the preprocessing stage users can trim adapters, untemplated additions and barcodes, move UMIs to header of reads and remove non-coding RNAs. Then users can choose whether they want to map reads to the genome and explore the alignments in GWIPS-viz and/or map reads to the transcriptome and explore Ribo-seq alignments in Trips-viz. Output formats are shown in blue.

The tool suites within RiboGalaxy underwent a rearrangement where each tool was redeveloped to support upgrades to the backend. Old and deprecated tools along with dependencies were either deleted, updated or replaced with alternatives and additional new functionalities have also been introduced. All the tools are now available in the Galaxy ToolShed enabling their incorporation into other Galaxy instances. The typical pipeline that users can run on RiboGalaxy is shown in Figure 1.

In the original version of RiboGalaxy, the *Preprocessing Suite* allowed users to trim adapters and untemplated additions via cutadapt^30^ and remove unwanted rRNAs with bowtie.^31^ In this update we also introduced a number of new tools to this suite. Users can perform data quality control using FastQC and to trim any untemplated additions as desired using a custom ‘Trim Sequences’ tool. RiboGalaxy currently provides pre-built rRNA and tRNA indices for six model organisms: *Homo sapiens,Mus musculus,Arabidopsis thaliana, Saccharomyces cereviae, Drosophila melanogaster, Escherichia Coli*. tRNA indices are a new addition to RiboGalaxy as only pre-built bowtie rRNA indices were provided at the time of the last update. Users can also supply their own rRNA/tRNA FASTA files for any organism not included in the list of pre-built indices.

Entirely new to RiboGalaxy is the *UMI and barcodes suite*. Since the initial RiboGalaxy release, the use of Unique Molecular Identifiers (UMIs) and barcode sequences has become much more popular. With the tool ‘UMIs to header’ users can move UMIs from a read sequence to header enabling PCR deduplication downstream. PCR deduplication functionality is now supported on RiboGalaxy via UMI-tools (‘UMI tools deduplicate’)^32^ which can be deployed on alignment files.

Transcriptome alignment is available under the *Trips-Viz (transcriptome mapping) branch suite* where alignment can be performed using bowtie with ‘Bowtie Transcriptome Alignment’. Pre-built transcriptome indices are now available for *Homo sapiens* (GENCODE v25, v28 and v39); *Mus musculus* (GENCODE M10, M14, M28); *Arabidopsis thaliana* (TAIR10); *Saccharomyces cerevisiae* (v3, v9); *Drosophila melanogaster* (dm3, dm6); *Escherichia Coli* (K12 ASM584v2). The provision of prebuilt indices speeds up the read alignment steps considerably and also minimises the amount of allocated storage used up by these processes.

The previous version of RiboGalaxy contained RiboTools and riboSeqR^33,34^ suites for exploring subcodon Ribo-Seq profiles, metagenes and triplet periodicity plots. Since then, the interoperability of all RiboSeq.Org resources has been improved and now downstream analysis is completely catered for by Trips-Viz. Apart from the aforementioned analysis, Trips-Viz also provides differential gene expression analysis, prediction of translated ORFs and identification of ribosome pause sites. RiboGalaxy now supports the production of the necessary read data and annotation file formats for upload to Trips-Viz. The ‘Create Trips-Viz annotation’ tool allows users to prepare their own custom transcriptome annotation database enabling them to carry out downstream analysis on transcriptomes not automatically supported by Trips-Viz. This tool takes a transcriptome annotation file (GTF/GFF3) and reference sequence file (FASTA), as well as a sample ‘Transcript ID’ and ‘Gene Name’ from the GTF/GFF3 files to help with file parsing. Optional ‘Pseudo UTR length’ is necessary when you use a transcriptome lacking annotated untranslated regions (UTRs). On the next step, ‘BAM to Sqlite’ function takes an organism annotation file in SQLite format from the previous step and a BAM file containing transcriptome alignments as inputs along with a one line description of the file being processed. This tool then produces a SQLite file containing information of the read alignments that is ready for submission to Trips-Viz. For the eight transcriptomes supported by RiboGalaxy we provide an organism annotation file in.sqlite format as a built-in option: *Homo sapiens* (GENCODE25, 28 and 39); *Mus musculus* (GENCODE M10, M14, M28); *Arabidopsis thaliana* (TAIR10); *Saccharomyces cerevisiae* (R64); *Escherichia Coli* (K12 ASM584v2). This makes it far easier for a user to proceed with their data interrogation within the RiboSeq.Org platform.

To ensure the quality of analysis and their data, the user can consider the following suggestions. After transferring a SQLITE file to Trips-Viz, the user can verify features typical to ribosome profiling analysis. Most of the footprints should align to CDS regions. Also ribosome profiling data typically exhibit a triplet periodicity which is manifested as an unequal distribution of footprint ends alignments across the three subcodon positions. Both features could be assessed by exploring a metagene profile; the footprints density at CDS should be greater than in UTRs. A pattern of triplet periodicity is expected in CDS but not UTRs. However, it should be noted that these features do not depend entirely on the data analysis and failure to observe them could be due to the data.

Genomic alignment and tools required for preparing files for upload to GWIPS-viz as custom tracks are available in the *GWIPS-viz (genomic alignment) branch suite*. ‘Bowtie Genome Alignment’ can be used to align reads to the genome using bowtie, then ‘Samtools sort’ will sort the file using the preset option (coordinate sorting). Next, ‘Create Ribosome Profiles’ takes a coordinate-sorted BAM file and corresponding genome reference as inputs and outputs a ribosome profile which then serves as input in ‘Convert a BED File to a bigWig’. In order to visualise genome alignments in GWIPS-viz using a compressed file format such as bigWig the file must be hosted at a web-accessible http, https or ftp link. The new ‘Generate Custom Track’ tool on RiboGalaxy produces a GWIPS-viz and UCSC compatible custom track file that links a bigWig file produced on RiboGalaxy to the custom track functionality in the browser without the need for external hosting. We also provide step-by-step guidelines of how to use RiboGalaxy tools, test files and a hands-on tutorials tailored to process it (see Data Availability section).

RiboGalaxy allows researchers to produce both genomic and transcriptomic alignments. When mapping Ribo-seq data on the genome, the user can explore translated regions genome-wide and find novel translated ORFs outside of annotated transcripts. Since genome-mapped Ribo-seq data can be visualised in the GWIPS-viz genome browser, the user can also supplement their samples with other types of data: Mappability tracks,^35^ conservation tracks (PhyloP^36^ and PhyloCSF,^37^ different transcriptome annotation tracks (including custom ones) and many other tracks that can be supplied into GWIPs-viz. Mapping ribosome profiling to annotated transcripts as in Trips-Viz helps to avoid mappability issues associated with exon-exon junctions occurring in genomic alignment. Visualising transcriptomic alignments in Trips-Viz allows the user to produce sub-codon profiles, where footprints supporting each frame are differentiated. Trips-Viz affords the user the possibility to directly compare their own data with the many published Ribo-seq and ancillary data hosted on Trips-Viz. For example, Trips-Viz also contains proteomics data which may provide orthogonal support for Ribo-seq based prediction of novel protein-coding regions. One disadvantage of transcriptome-based alignment approach however, is the imposition of keeping pace with regularly updated gene annotations.

### Published workflows

We updated published workflows for genome and transcriptome mapping that are available under the Shared Data tab. At present, RiboGalaxy provides two published workflows that facilitate the processing of Ribo-Seq data for RiboSeq.Org’s visualisation tools in a single execution (*Trips_viz_pipeline* and GWIPS*_viz_pipeline*). Each tool is configured to use appropriate default parameters for this data type. Prior to execution, however, users can update any parameter as they wish.

### Data types that can be processed

Since the development of the Ribo-Seq technique and the original RiboGalaxy instance, different modifications of ribosome profiling have been developed and become widely used over the past decade. In RiboGalaxy the user can process not only regular elongating ribosome profiling in different species, but also Translation Initiation sequencing or TI-seq (e.g. data from^38^). TI-seq can be processed in the same manner as standard Ribo-Seq. In case of TCP-seq,^39^ the whole pipeline is also the same except when the user wants to upload SQLite to Trips-viz, they need to select ‘File type’ - ‘Other’ and put the name: ‘TCP-seq’. For bacterial Ribo-seq (e.g. see^40^) it is important to note that the use of Micrococcal nuclease (see protocol^41^) significantly decreases triplet periodicity. Thus even though the subcodon profiles can be visualised in Trips-viz, they are not as informative as for eukaryotic samples. We provide example files and corresponding tutorials in the Data Availability Section.

### Comparison with similar resources

A number of tools also offer Ribo-Seq data processing and analysis through a web interface, however, a smaller number offer end-to-end processing. For example, MappingQC,^42^ RiboDiff^43^ and RIVET^44^ provide either Galaxy or R Shiny interfaces for their Ribo-Seq related applications but for each of these tools a some level of preprocessing is required. It is also worth noting that RiboTools^33^ is a set of Galaxy tools for qualitative Ribo-Seq analysis but there is no dedicated public Galaxy interface for these tools. End-to-end translated ORF detection via a Galaxy interface is offered through the PROTEOFORMER^42^ pipeline.

End-to-end Ribo-Seq data processing and analysis within a web browser similar to that offered by RiboGalaxy (coupled with GWIPS-viz and Trips-Viz) is also offered by RiboToolKit^45^ and Translatome Workbench.^46^

In terms of input data, RiboGalaxy and Translatome workbench take raw data files and carry out the preprocessing steps for the user. RiboToolKit requires that the inputted FASTQ files have their adapters removed and that pre-alignment QC has already been handled. RiboGalaxy and Translatome workbench support pre-alignment quality control with FastQC and removal of user-defined adapters. Translatome workbench offers a choice of FastP or Trimgalore for adapter removal whereas RiboGalaxy uses Cutadapt by default. RiboGalaxy is the only tool of these three to support barcode and UMI handling or removal of untemplated additions.

Keeping in line with the standard data processing workflows of GWIPS-viz and Trips-Viz, RiboGalaxy offers bowtie for read alignment. Maintaining consistent read alignment algorithms between datasets on these resources minimises non-biological variation between samples. Bowtie is a non-splice aware aligner meaning it will not attempt to map reads across splice junctions. This leads to a characteristic drop off of mapped reads around junctions when alignment is carried out against the genome. However, using bowtie to align directly to the transcriptome results in a more uniform profile relative to one produced by a splice aware aligner (eg. STAR) when mapping to the genome (which is recommended) and transforming genomic coordinates to transcriptomic ones as short Ribo-Seq reads are less likely to map across splice junctions without ambiguity.

For the removal of non-coding RNA from each sample RiboToolKit also uses bowtie. However, RiboToolKit employs STAR for alignment to the reference. Translatome Workbench offers splice aware aligners, namely STAR, TopHat2 and HISAT2 for reference alignment and does not support the removal of non-coding RNA.

Extensive downstream analysis in the browser is possible within RiboSeq.Org using Trips-Viz rather than RiboGalaxy. The improved interoperability of these resources makes this transition relatively straightforward. Translatome Workbench and RiboToolKit offer these steps within their workflows directly.

RiboSeq.Org offers extensive post-alignment quality control *via* Trips-Viz. Users can access read mapping statistics, view metagene profiles, generate read length specific triplet periodicity plots and look at the distribution of reads across the mRNA all within the browser. RiboToolKit offers a similar variety of plots in its final report, while the post-alignment QC outputs of Translatome Workbench vary depending on the user chosen application for post alignment QC. They offer QC functionality from Ribo-seQC, RiboWaltz, Plastid, RiboTaper and Ribo-TISH QC.

Translatome Workbench also offers a selection of publicly available ORF detection algorithms (Ribo-TISH, RiboCode, RiboTricer, and PRICE) while RiboToolkit employs the RiboCode algorithm. Trips-Viz offers its own novel ORF calling module. Similarly, all three platforms offer the generation of gene count matrices and differential expression analysis. However, gene set enrichment analysis is only offered by RiboToolKit.

### Future plans

We adopted a more recent Galaxy framework^47^ to build this updated RiboGalaxy. We plan to continue developing RiboGalaxy by adding more Ribo-Seq related tools and also expand computational resources. E.g. Once we add more resources (RAM and storage) to the virtual machine, we will be able to incorporate STAR^48^ as an alternative tool for genome alignment since it performs faster and performs better in mapping reads across exon-exon junctions thus making Ribo-Seq coverage smoother than the current genome alignment implementation.

## Materials and Methods

RiboGalaxy runs on Ubuntu 20.04 LTS with the help of Ansible configuration management based on Galaxy release 21.09. In this version we added a dedicated data processing section for Trips-Viz - a transcriptome browser for visualisation and analysis of Ribo-Seq data. We migrated python-dependent tools from Python 2 to Python 3. Old versions of packages were updated (current versions: bowtie 1.2, samtools 1.15, cutadapt 3.7, pysam 0.19.0, bedtools 2.30.0). Instead of using a local toolshed, we wrapped our tools into modules recognised by Galaxy and submitted them to the Galaxy ToolShed.^49^ Config and tool files are available at https://github.com/riboseqorg/RiboGalaxy. We configured RiboGalaxy to run jobs using Singularity containers^50^ provided by the BioContainers community.^51^ Slurm was used as a distributed resource management system.

## Figures and Tables

**Table 1:** List of updates in comparison to the previous version of RiboGalaxy (published in 2016).

Features	RiboGalaxy as of 2016	RiboGalaxy as of 2023	Significance of change
Configuration	Primarily Manual	Ansible Based	RiboGalaxy’s configuration is now easily replicable
Containerisation	None	Jobs are Run in Singularity containers	Reliable control over the environment that jobs are run in
Tool versions	Standard for 2016	Up-to-date	Adds features and reduces bugs
Toolshed tools	several tools available in toolshed	All tools available in toolshed	Anyone with a running Galaxy instance can add RiboGalaxy tools
Pre-Processing	Adapter trimming, untemplated addition removal, rRNA removal	Added UMI and barcode handling and rRNA and **t**RNA removal	Expanded options in line with current methodologies
Quality Control	FASTQC, metagene and triplet periodicity plots with RiboSeqR	FASTQC, Connect with Trips-Viz for metagene, read length distribution triplet periodicity, and more	Post-alignment QC is now handled by the extensive tools available in Trips-Viz
Interoperability with RiboSeq.Org	Produced file types compatible with GWIPS-viz	Produces file types compatible with GWIPS- viz and Trips-Viz. Added tools to help link between RiboGalaxy and other resources	Focus of these resources has changed to emphasise the combined use of all RiboSeq.Org resources
Built-in Resources	rRNA and Genome Bowtie indices	rRNA, tRNA, Genome and Transcriptome bowtie indices. Trips-Viz annotation files for 8 transcriptomes	Expanded diversity of builtin resources reducing processing time and storage requirements for each user
Workflows	One workflow per species using genomic alignments only	Maintained support for all species but restructured with a focus on producing files for GWIPS-viz, Trips-Viz or both	Increased the offerings in terms of preconfigured workflows

## Data Availability

Here we deposited test datasets for testing RiboGalaxy and hands-on tutorials in https://www.dropbox.com/sh/6ez3vba1u18n80q/AAB6me63rU3CUd_k0Q2vlBUja?dl=0. Source code is available at https://github.com/riboseqorg/RiboGalaxy. General tutorials explaining how to use tools are available at https://jackcurragh.github.io/RiboGalaxy-Tutorials/.

## Data Availability

Code have been deposited in github.
